# Retrocaval ureter and contra lateral renal agenesis – a case report and review of literature

**DOI:** 10.1590/S1677-5538.IBJU.2015.0549

**Published:** 2016

**Authors:** Felix Cardoza, C. K. Shambhulinga, A. T. Rajeevan

**Affiliations:** 1 Department of Urology, Government Medical College, Calicut, Kerala, India

**Keywords:** Retrocaval Ureter, Hereditary renal agenesis [Supplementary Concept]

## Abstract

Associated congenital anomalies are seen in 21% of retrocaval ureter patients; among them, associated contralateral renal agenesis is a very rare entity. We report one such case of right circumcaval ureter with left renal agenesis, diagnosed after febrile UTI. Surgical correction with uretero-ureterostomy was successful. In literature very few such cases are reported and only one case with renal failure was reported. Unilateral renal agenesis cases complicated by associated such anomalies need definitive management and lifelong clinical monitoring to diagnose and prevent chronic kidney disease.

## INTRODUCTION

Retrocaval ureter, a misnomer, correct denomination circumcaval ureter, was first reported by Hochstetter in 1893 (1). Even though its incidence is about 1 in 1100 according to autopsy series, around 200 cases are reported in the literature. Circumcaval ureter associated with contra-lateral renal agenesis is a very rare presentation, very few cases reported in the literature. These cases need surgical correction and close clinical follow-up.

**Figure 1:**
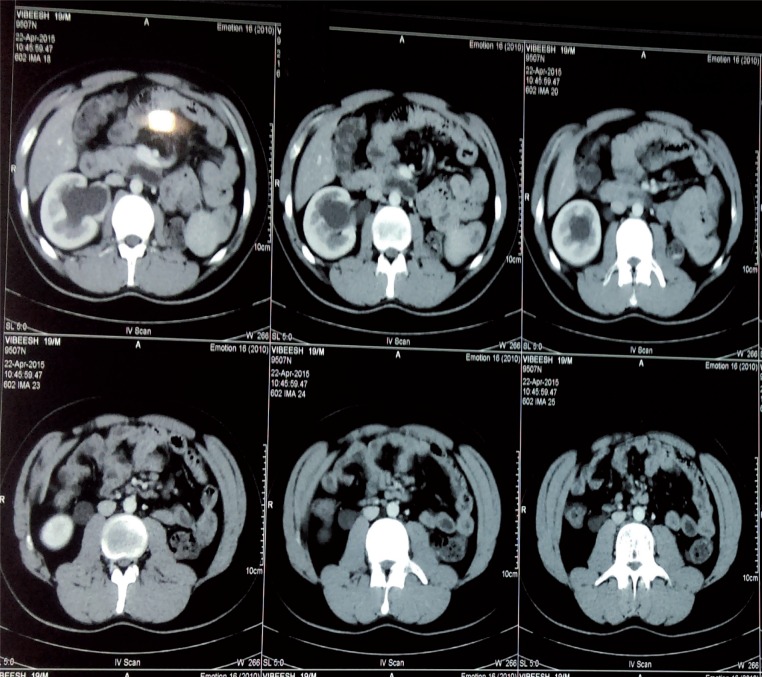
CT scan image, shows absent left kidney and right hydronephrosis and hydroureter up to L3 vertebra at which level it passes behind IVC.

**Figure 2:**
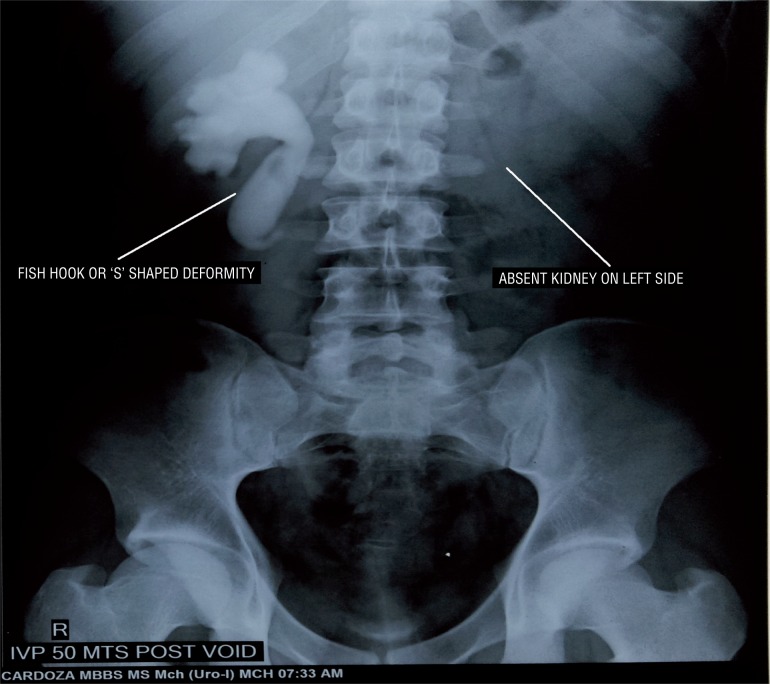
IVP 50 min post void image, shows Fish hook or ‘S’ shaped deformity of right ureter with absent uptake/excretion of dye on left side.

**Figure 3:**
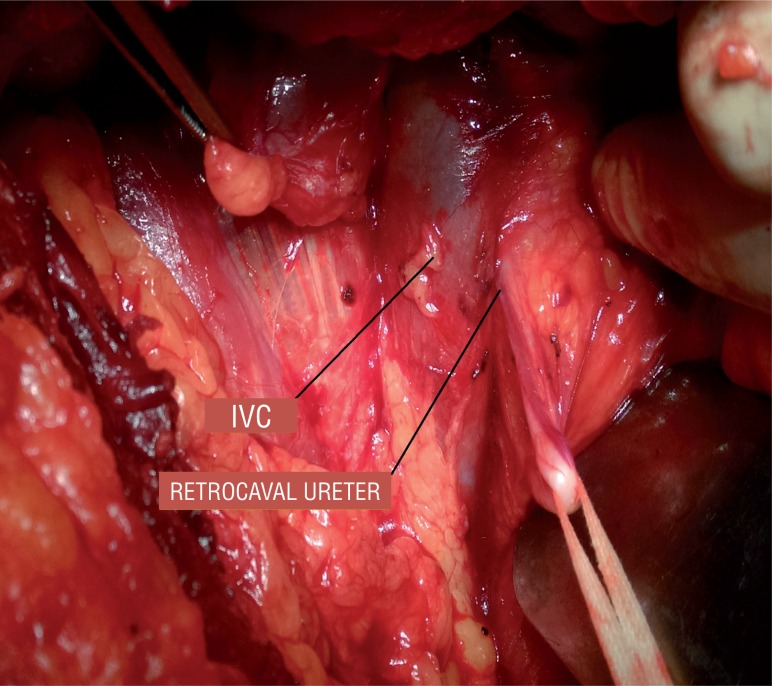
Intra operative picture showing circumcaval ureter.

Case HTistory: 19 year-old male patient presented with fever with chills for 1 day. Patient had an unremarkable examination except for right renal angle tenderness. Urinalysis showed 8-10 piuria cells and urine culture was negative for any bacteria. His renal function test was normal (Blood urea-18 and serum creatinine-0.9). Ultrasonography revealed non visualised left kidney in renal fossa with normal sized right kidney with hydronephrosis with antero-posterior diameter at pelvis of 20mm, with internal echoes and proximal hydroureter for about 6cm from PUJ. Computed tomography shown absent left kidney and normal sized right kidney with hydronephrosis and dilated ureter up to L3 vertebra where the ureter was seen passing behind the inferior vena cava with features of pyelonephritis such as striated nephrogram (Figure-1). Intravenous urography showed Fish hook or ‘S’ shaped deformity (Figure-2). Patient was diagnosed as right solitary functional kidney with retrocaval ureter with pyelonephritis. Patient was treated for pyelonephritis and he underwent right uretero-ureterostomy over 6Fr/26cm double J stent; retrocaval portion of the ureter was atretic and was excised (Figure-3). Patient had an uneventful recovery. On follow-up patient is doing well.

## DISCUSSION

Retrocaval ureter is a rare congenital anomaly, which results from abnormality in the development of the inferior vena cava (IVC). Normally the fetal veins such as the vitelline vein, subcardinal vein and sacrocardinal vein undergo sequential development, anastomosis and regression to form IVC. Vitelline vein develops into pre renal segment of IVC, subcardinal vein forms renal segment of IVC and sacrocardinal vein forms post renal segment of IVC. Pre ureteral vena cava or retrocaval ureter develops when the renal segment of IVC develops from the abnormal persistence of right posterior cardinal vein (which fails to atrophy during development), which lies ventral to ureter.

These patients usually present in their third or fourth decade of life (2). They may be asymptomatic or may present with urinary tract infection, hematuria, flank pain, calculus disease or renal failure. This anomaly is diagnosed on IVU and confirmed by CT scan or MRI scan.

According to Bateson and Atkinson classification, retrocaval ureter is classified into two types. Type I or low loop type, accounts for 90% of the cases: here ureter crosses behind the IVC at the level of L3 vertebra, and has a ‘S’ or Fish hook shaped deformity of the ureter on IVP, has marked hydronephrosis in up to 50% of the patients. In type II or high loop type, less common, accounts for 10% of the cases. Here renal pelvis and upper ureter lies horizontally, the retrocaval segment of the ureter is at the same level of the renal pelvis. On retrograde pyelogram the involved ureter looks like ‘Sickle’ shaped, and patient may have mild hydronephrosis. Main causes for hydronephrosis in these cases are because of stenosis, adhesion of the retrocaval segment and torsion (3).

Associated congenital anomalies are seen in 21% of retrocaval ureter patients which include horseshoe kidney, ectopic or malrotated opposite kidney, contra lateral renal agenesis, hypospadias, ureteropelvic junction obstruction (UPJO), congenital lack of vas deferens, agenesis of uterus and vagina, yolk sac tumour, myelomeningocele, variations of inferior vena cava, oesophageal atresia, cardiovascular anomalies such as situs inversus, brachial arch syndrome, Turner syndrome, and imperforate anus (4). Other disorders that have been reported to be associated with retrocaval ureter are renovascular hypertension, retroperitoneal fibrosis and carcinoma of the ureter. These patients usually need surgical correction such as ureteropyelostomy or uretero-ureterostomy if symptomatic.

In our case, patient presented with right pyelonephritis, he had right retrocaval ureter with contra lateral renal agenesis. Based on the investigation findings, he had type I retrocaval ureter. Patient underwent right uretero-ureterostomy over DJ stent after excising the atresic retrocaval segment. Patient had uneventful recovery.

There are two such cases reported in the literature, one with renal failure, the other with associated agenesis of uterus and vagina and imperforate anus. 10-20% of patients with URA (unilateral renal agenesis) may need dialysis by the age of 30 years if they are associated with other genitourinary anomalies such as PUV (posterior urethral valve), UPJO, retrocaval ureter etc. (5). If a patient with URA has associated retrocaval ureter, then the patient is even more prone for renal failure from contributing complications such as obstruction, pyelonephritis etc.

## CONCLUSIONS

Patients with retrocaval ureter are looked for other associated congenital genitourinary and other anomalies carefully. Whenever associated contralateral renal agenesis is noted, they need a prompt surgical correction for retrocaval ureter and lifelong clinical monitoring and follow-up to prevent and early diagnose acute or chronic renal failure.

## References

[1] Hochstetter F (1893). Beitrage zur entwicklungsgeschichte des venen-systems der amnioten: III. Sauger Morph Jahrb.

[2] Feldman SL, DiMarco ER, Tencer T, Ross LS (1982). Retrocaval ureter: radiographic techniques directing surgical management. Br J Urol.

[3] Bateson EM, Atkinson D (1969). Circumcaval ureter: a new classification. Clin Radiol.

[4] Perimenis P, Gyftopoulos K, Athanasopoulos A, Pastromas V, Barbalias G (2002). Retrocaval ureter and associated abnormalities. Int Urol Nephrol.

[5] Sanna-Cherchi S, Ravani P, Corbani V, Parodi S, Haupt R, Piaggio G (2009). Renal outcome in patients with congenital anomalies of the kidney and urinary tract. Kidney Int.

